# SETD1A-dependent EME1 transcription drives PARPi sensitivity in HR deficient tumour cells

**DOI:** 10.1038/s41416-025-02963-0

**Published:** 2025-02-24

**Authors:** Ellie Sweatman, Rachel Bayley, Richad Selemane, Martin R. Higgs

**Affiliations:** Department of Cancer and Genomic Sciences, School of Medical Sciences, College of Medicine and Health, University of Birmingham, Birmingham, UK

**Keywords:** Cancer therapeutic resistance, Methylation

## Abstract

**Background:**

Cells deficient in DNA repair factors breast cancer susceptibility 1/2 (BRCA1/2) or ataxia-telangiectasia mutated (ATM) are sensitive to poly-ADP ribose polymerase (PARP) inhibitors. Building on our previous findings, we asked how the lysine methyltransferase SETD1A contributed to PARP inhibitor-mediated cell death in these contexts and determined the mechanisms responsible.

**Methods:**

We used cervical, breast, lung and ovarian cancer cells bearing mutations in *BRCA1* or *ATM* and depleted SETD1A using siRNA or CRISPR/Cas9. We assessed the effects of the PARPi Olaparib on cell viability, homologous recombination, and DNA repair. We assessed underlying transcriptional perturbations using RNAseq. We used The Cancer Genomics Atlas (TCGA) and DepMap to investigate patient survival and cancer cell characteristics.

**Results:**

Loss of SETD1A from both *BRCA1*-deficient and *ATM*-deficient cancer cells was associated with resistance to Olaparib, explained by partial restoration of homologous recombination. Mechanistically, SETD1A-dependent transcription of the crossover junction endonuclease EME1 correlated with sensitivity to Olaparib in these cells. Accordingly, when SETD1A or EME1 was lost, *BRCA1* or *ATM*-mutated cells became resistant to Olaparib, and homologous recombination was partially restored.

**Conclusions:**

Loss of SETD1A or EME1 drives cellular resistance to Olaparib in certain genetic contexts and may help explain why patients develop resistance to PARP inhibitors in the clinic.

## Introduction

Poly-ADP-ribose polymerase (PARP) inhibitors (PARPi) were first identified as potential targeted cancer treatments for tumours with deficiencies in the homologous recombination (HR) pathway of double strand break (DSB) repair [1]. This is exemplified by the exquisite sensitivity of cells bearing mutations in breast cancer susceptibility 1 (*BRCA1*) or 2 (*BRCA2*) to PARP inhibition [2]. To date, four different PARP inhibitors (Olaparib, Rucaparib, Talazoparib and Niraparib) have received clinical approval for the treatment of subsets of breast [3], ovarian [4], pancreatic [5] and prostate cancer [6, 7]. This ranges from widespread use alongside conventional chemotherapies to a more limited use in metastatic settings after failure of other treatment options.

Unfortunately, despite the success of PARPi in the clinic, patient prognosis is frequently hampered by the development of resistance. Indeed, approximately 40% of metastatic breast cancer patients harbouring germline *BRCA1/2* mutations fail to respond to Olaparib treatment [3], suggesting widespread pre-existing resistance. This emphasises the need to further characterise PARPi resistance mechanisms, and to identify strategies to overcome resistance.

From extensive in vitro studies, several mechanisms of resistance to PARPi have been described e.g. [8–10]. These include hypomorphic secondary mutations in *BRCA1/2* that partially restore function, perturbation of PARP1/2 or PARG expression, or re-wiring of the DNA damage response. In this latter case, alterations in fork protection, use of alternative error-prone pathways such as alt-EJ or the repair/prevalence of ssDNA gaps all cause PARPi resistance in BRCA-deficient cells. One of the most well-studied resistance mechanisms to PARPi is the loss of pro-NHEJ factors, which drives partial restoration of functional HR. This includes loss of 53BP1 [11, 12] or inactivation of the downstream effectors RIF1, REV7 and Shieldin [13–18]. Loss of this pathway and concomitant restoration of HR has been observed in mouse models of *BRCA1* deficiency, in patient-derived xenograft models, and in patients [12, 19–21].

Recently our lab identified the lysine methyltransferase SET domain containing protein 1 A (SETD1A) as an anti-resection factor that suppresses BRCA1-mediated DNA end-resection, and which is vital for RIF1-dependent NHEJ [22]. Loss of SETD1A abrogates RIF1 chromatin binding and compromises NHEJ, facilitating BRCA1-independent DNA end resection in BRCA1-deficient cells. This enables partial restoration of HR and resolution of PARPi-induced DSBs, conferring PARPi resistance in vitro [22, 23].

Whilst much research has focused on the impact of PARPi in BRCA-deficient cells, the clinical efficacy of PARPi extends beyond BRCA1/2 to include mutations in other DNA repair proteins, such as the RAD51 recombinase, RAD51 paralogs C and D (RAD51C/D), excision repair cross-complementation group 1 (ERCC1), partner and localiser of BRCA2 (PALB2), and ataxia-telangiectasia mutated (ATM) [24–27]. ATM is a phosphatidyl inositol 3 kinase-like kinase that plays a key role in initiating DSB repair, and is a known cancer susceptibility gene that is mutated in 5% of all cancers, including 40% of mantle cell lymphomas, 20% of colorectal cancers and 10% prostate and lung cancers [28, 29]. Early studies demonstrated that depletion of ATM induces PARPi sensitivity, indicating that *ATM*-mutated cancers might benefit from PARPi therapy [30]. Subsequently, *ATM*-deficient lung, gastric, prostate, brain and lymphoid tumours have all been shown to exhibit PARPi sensitivity [31–35]. However, the use of ATM as a clinical biomarker for PARPi sensitivity is still limited, and trials in non-small cell lung cancer in which *ATM* is frequently mutated have proved inconclusive [36]. Moreover, whilst mechanisms of PARPi resistance are well studied in the context of BRCA-deficiency, it is unclear whether these apply to other HR-deficient contexts including *ATM* deficiency.

In this study we ask whether SETD1A-induced PARPi resistance is specific to BRCA1-deficiency or whether it could be broadly applicable to other HR deficient cancers, focusing on those deficient in *ATM* or *BRCA2*. To do so, we assessed cellular survival and DNA repair kinetics in response to Olaparib in a range of HR-deficient cell lines in the presence/absence of SETD1A. Due to its role as a lysine methyltransferase that targets H4K4, we also utilised CRISPR/Cas9 to knock out *SETD1A* and performed RNA sequencing analysis to identify any transcriptional mechanisms that underpin SETD1A-induced PARPi resistance.

Our findings demonstrate that loss of SETD1A also drives PARPi resistance in *ATM*-deficient cancer cells. Interestingly, SETD1A-dependent transcription of the crossover endonuclease EME1 underpins PARPi sensitivity in vitro, and depletion of EME1 from HR-deficient cells drove resistance to Olaparib. SETD1A and EME1 expression and/or activity could therefore offer a potential new combinatorial biomarker to help stratify patients who will benefit from PARPi therapy, reducing the heterogenous response observed in the clinic and improving patient outcomes.

## Materials and methods

### Cell lines and culture

All cell lines were maintained at 37 °C and 5% CO_2_ and passaged by trypsinisation at sub-confluency. HeLa (CVCL_0030; ATCC), HeLa-H3 GFP (WT and K4A) [37, 38], MCF-7 (ATCC; CVCL_ 0031) and A549 (ATCC; CVCL_0023) cell lines were cultured in Dulbecco’s modified Eagle’s media (DMEM), supplemented with fetal calf serum (FCS) (10% v/v). H1395 cell lines (ATCC; CVCL_1467) were cultured in RPMI-1640 media supplemented with FCS (10% v/v), 1 mM sodium pyruvate and 10 mM HEPES. SKOV3 (ATCC; CVCL_0532) cell lines were cultured in McCoy 5 A (Modified) Medium supplemented with FCS (10% v/v). UWB1.289 (ATCC; CVCL_B079) cell lines were cultured in RPMI-1640 media containing Mammary Epithelial Growth Medium (Lonza) (50% v/v) supplemented with FCS (3% v/v) and L-glutamine (50% v/v). HCC-1937 (DSMZ; CVCL_0290) cell lines were cultured in RPMI-1640 media containing L-glutamine supplemented with FCS (10% v/v). HeLa Kyoto iCas9 [22] cells transduced with a lentivirus expressing sgRNA targeting human SETD1A (AGAGCCATCGGAAATTTCCG) or a non-targeting control were obtained from Dr Simon Boulton. They were cultured in DMEM supplemented with tetracycline-free FCS (10% v/v). Cas9 expression was induced with 1 µg/ml doxycycline for 48 h. All media and additives were from Life Technologies unless specified. Cells were tested for mycoplasma contamination monthly and verified by STR profiling annually.

### siRNA transfections

Cells were transfected with SMARTpool siRNAs (Horizon Discovery) at a final concentration of 100 nM using Oligofectamine (Life Technologies) following the manufacturer’s instructions. siRNA targeting LacZ (Horizon Discovery; CGUACGCGGAAUACUUCGdTdT) was used as a control.

### Antibodies

The following antibodies were used: RAD51 (Millipore; Cat# PC130), SETD1A (Bethyl; Cat# A300-289A), ATM (Santa Cruz; sc-53173), BRCA1 (Santa Cruz; Cat # sc-6954), BRCA2 (Santa Cruz; sc-6954), PARP1 (Santa Cruz; sc-74470), EME1 (Santa Cruz; sc-53275), KAP1 (Bethyl; A300-274A), P-KAP1 Ser 824 (Bethyl; A300-767A), β-tubulin (Cell Signalling; 86298S), histone H3 (Abcam; ab1791), CENPF (BD; 610768), Alexa-Fluor anti-rabbit 488 (ThermoFisher; A11070), Alexa-Fluor anti-mouse 594 (ThermoFisher; A11032), Alexa-Fluor anti-rabbit 594 (ThermoFisher; A-21207), Anti-rabbit HRP (Agilent; P0399), Anti-mouse HRP (Agilent; P0447).

### Clonogenic survival assays

Appropriate cell lines were transfected with indicated siRNAs and/or treated with 1 µM AZD0156 (SelleckChem) for 24 h where indicated. Seventy-two hours post transfection/treatment, cells were seeded at low density and treated with the denoted concentrations of Olaparib (AZD2281; Selleckchem) for 3 days. Olaparib was then replaced every 3 days for a total of 10 days. Colonies were then stained with 0.5% crystal-violet (Sigma-Aldrich) in ddH_2_O and counted, or colonies were resolubilised using 1% SDS solution and absorbance measured at 595 nm. Data are expressed as percentage survival normalised to untreated controls for each condition.

### Immunofluorescence

Permeabilisation, fixation and staining of cells on glass coverslips was carried out as previously described [22]. Images were acquired with a Nikon E600 Eclipse equipped with a 60x oil lens and Nikon Elements software, and foci numbers analysed using Image J (NIH). For each independent experiment >100 nuclei were counted per condition, and slides were blinded before enumeration.

### Homologous recombination assay

Homologous recombination was measured using a CRISPR-based LMNA assay as previously described [39]. Briefly, HeLa cells were transfected with siRNA, seeded onto glass coverslips and co-transfected with plasmids encoding Cas9, a gRNA targeting LMNA, a donor plasmid encoding clover-LMNA, and a plasmid expressing RFP. Forty-eight hours later, coverslips were fixed using 4% paraformaldehyde and cells permeabilised in PBS supplemented with Trition-X-100 (0.5% v/v) for 5 min at room temperature. Coverslips were then blocked as above before being mounted onto slides and imaged as described above. Homologous recombination activity was quantified by enumerating clover-LMNA positive cells and normalising to RFP transfection efficiency. For each independent experiment >100 nuclei were counted per condition.

### Metaphase spreads

Cells were transfected with siRNA then treated with 10 µM Olaparib for 24 h. Three hours prior to harvesting cells were treated with 0.1 µg/ml Colcemid (ThermoFisher Scientific). Cells were harvested by trypsinisation, incubated in prewarmed 0.075 M KCl for 10 min, and fixed in ice cold fixative solution (3:1 methanol/acetic acid). Metaphases were dropped onto glass slides pre-soaked in fixative and placed on a heat block at 80 °C for 1 min, before being allowed to dry overnight. Slides were mounted with Duolink in situ mounting medium containing DAPI (Sigma-Aldrich) and imaged as above using a 100x oil lens. Radial chromosomes were enumerated using ImageJ software, and at least 50 metaphases were counted per condition for each independent experiment. Images were blinded before enumeration.

### Chromatin fractionation

HeLa Kyoto iCas9 Cells were harvested by trypsination and lysed in chromatin fractionation buffer (20 mM Tris-HCl, 100 mM NaCl, 5 mM MgCl_2_, 10% glycerol, 0.2% IGEPAL CA-620, 0.5 mM DTT, protease inhibitor cocktail) on ice for 15 min. Lysates were clarified at 200 × *g* for 5 min at 4 °C, supernatant was removed to form a ‘soluble fraction’ and the chromatin pellet was washed and resuspended in fractionation buffer before two cycles of sonication at 10% power for 10 s.

### Western blotting

Immunoblotting of whole cells extracts (WCE) or cellular fractions was performed as previously described [22, 37].

### RNA sequencing

HeLa Kyoto iCas9 expressing SETD1A gRNA were transfected with control, ATM or BRCA1 siRNA as described above and Cas9 expression induced with doxycycline where appropriate. Total RNA was extracted from cell pellets using the RNeasy kit (Qiagen) with on-column DNase digest, and RNA quality confirmed using TapeStation (Aligent Technologies). mRNA was isolated using NEBNext Poly(A) mRNA magnetic isolation module and sequencing libraries were created using NEBNext Ultra II direction RNA library prep kit for Illumina (New England Biolabs). DNA libraries were validated with TapeStation (Aligent Technologies) and quantified using Qubit 2.0 Fluorometer (ThermoFisher Scientific), before being pooled and sequenced on Illumina NovaSeq 6000. Raw sequencing data was uploaded to the Galaxy web platform and analysed using the public server at https://usegalaxy.eu/ [40]. Sequencing data generated in this study have been deposited in the Expression Omnibus (GEO) database under accession code GSE271430.

### Quantitative polymerase chain reaction (qPCR)

cDNA was synthesised from 2 µg RNA (see above) using a High-Capacity cDNA Reverse Transcriptase kit (Applied Biosciences). cDNA was then quantified by TaqMan gene expression array (Applied Biosystems) with a Quantstudio 5 PCR system, and mRNA expression normalised to levels of GUS1B.

### Cancer genomics analysis

cBioPortal (https://www.cbioportal.org/) [41, 42] was used to access genomic data from TCGA lung adenocarcinoma, ovarian serous cystadenocarcinoma and TCGA and Metabric breast carcinoma datasets. Log2 mRNA expression of indicated genes was obtained from RNA-seq datasets. Patients were stratified based on SETD1A mRNA expression: Low= homozygous SETD1A deletion or mRNA expression <=-2 SD below mean; High= SETD1A mRNA expression >2 SD above mean. For Kaplan-Meir survival analysis *ATM*-, *BRCA1*- or *BRCA2*-deficient cancer patients were selected (defined as homozygous deletion, mutation, or mRNA expression <=-2 SD below mean) and clinical data downloaded. Patients were then stratified by low (bottom 50% mRNA expression) or high SETD1A (upper 50% mRNA expression) gene expression. RNA-seq, drug sensitivity and mutation profiles from breast, ovarian and lung cancer cell lines were obtained from DepMap (https://depmap.org/portal/) [43].

### ChIP-Seq analysis

Setd1a ChIP-Seq data from mouse embryonic stem cells was downloaded (GEO: GSE93538) [44]. Reads were aligned to mouse mm10 genome using Bowtie2, and peaks called using EaSeq. Plots were created in IGV Genome Browser for the selected genes.

### Statistical analyses and data reproducibility

For in vitro assays, assuming a significance level of 5% and 90% power, at least 3 independent experiments/group were required to detect a difference between conditions. For analyses of patient survival, a minimum of 5 samples were required (90% power and a significance level of 5%), but sample size was limited by data availability. The normality, variance, descriptive statistics and statistical significance of data was assessed using GraphPad Prism 10. For all in vitro experiments, *n* = three independent biological repeats, and the mean ± SEM is denoted unless indicated. For colony survival experiments, *n* = three independent biological repeats carried out in triplicate. Means were compared using either two-tailed unpaired Students *t* test, one-way ANOVA or two-way ANOVA with post-hoc Tukeys correction for multiple comparisons as indicated in the figure legends. Kaplan–Meir survivals were analysed using a Log-rank (Mantel-Cox) test. In all cases * = *P* ≤ 0.05, ** = *P* ≤ 0.01, *** = *P* ≤ 0.001, **** = *P* ≤ 0.0001.

## Results

### Loss of SETD1A reduces the sensitivity of ATM-deficient cells to Olaparib

Our previous work demonstrated that loss of the lysine methyltransferase SETD1A decreased the sensitivity of *BRCA1-*deficient cells to Olaparib or Talazoparib [22], which we attributed to partial restoration of HR in the absence of SETD1A/H3K4me3, suggesting a new epigenetic mechanism of PARPi resistance [23].

**Fig. 1:**
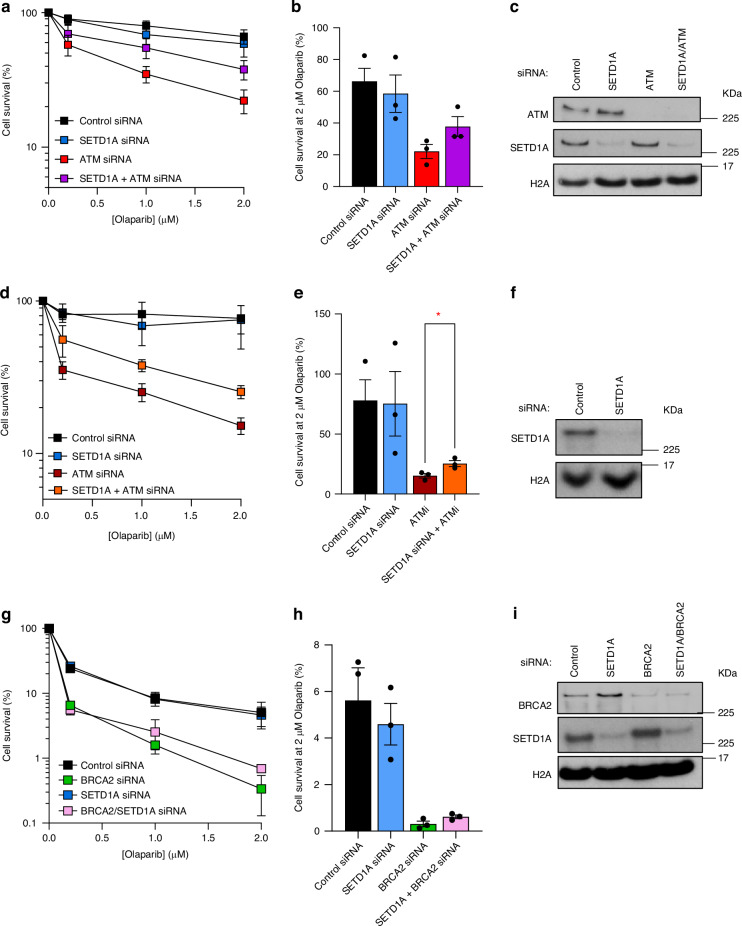
Loss of SETD1A reduces sensitivity of ATM-deficient cells to Olaparib **a**, **b** HeLa cells were transfected with the indicated siRNAs and left to form colonies under continual exposure to Olaparib at the denoted doses for 10 days. **b** denotes relative cell survival at 2 µM Olaparib. **c** Whole cell extracts of cells from A were analysed by immunoblotting using the indicated antibodies. **d**, **e** HeLa cells were transfected with the indicated siRNA, left for 48 h, and treated with 1 µM of ATM inhibitor AZD0156. Cells were then left to form colonies for 10 days under continual exposure to Olaparib at the denoted doses. **e** denotes relative cell survival at 2 µM Olaparib. **f** Whole cell extracts of cells from D were analysed by immunoblotting using the indicated antibodies. **g**, **h** MCF7 cells were transfected with the indicated siRNAs and left to form colonies under continual exposure to Olaparib at the denoted doses for 10 days. **h** denotes relative cell survival at 2 µM Olaparib. **i** Whole cell extracts of cells from G were analysed by immunoblotting using the indicated antibodies. Data points in all cases represent mean ± SEM from three independent biological repeats. * = *P* < 0.05 as determined by an unpaired two-tailed Student’s *t* test.

Multiple contexts of HR deficiency outside BRCA1 drive synthetic lethality with PARP inhibition, including alterations in *BRCA2*, *RAD51* and *ATM* [24, 25, 27, 45]. However, the resistance mechanisms relevant to these other HR-deficient contexts are not well understood. To gain further insights into these mechanisms, we first assessed whether loss of SETD1A conveyed resistance to PARP inhibitors in the absence of ATM activity. To this end, we assessed the impact of Olaparib treatment on cells depleted of ATM and/or SETD1A (Fig. 1a–c and Fig. S1A). As expected, knockdown of ATM resulted in sensitivity to Olaparib treatment, in keeping with its role in promoting HR, whilst loss of SETD1A alone had no effect. Interestingly, the sensitivity of *ATM*-deficient cells to Olaparib was partially lost upon co-depletion of SETD1A. These findings were further validated in cells treated with an ATM inhibitor, AZD0156 (Fig. 1d–f and Fig. S1B).

PARP inhibitors are also approved in the treatment of *BRCA2*-deficient tumours: however, mechanisms of PARPi resistance differ substantially compared to cells lacking BRCA1 [8, 46]. For example, loss of pro NHEJ factors such as 53BP1 cannot rescue HR activity and induce PARPi resistance in *BRCA2*-mutated cells [11]. In keeping, no restorative effect on HR was observed upon SETD1A depletion in MCF-7 cells lacking BRCA2, demonstrating that SETD1A-mediated PARPi resistance is not universal across HR-deficient cell lines (Fig. 1g–i). Together, these findings demonstrate that, in addition to *BRCA1*-deficient cells, sensitivity to PARP inhibition caused by loss of ATM function or its kinase activity can also be alleviated by depletion of SETD1A.

### SETD1A depletion partially restores HR in ATM-deficient cells

In *BRCA1*-deficient cells, loss of pro-NHEJ factors such as 53BP1, RIF1, REV7-Shieldin, and the lysine methyltransferase SETD1A and its cofactor BOD1L restore HR activity [13–18, 22, 47]. We therefore hypothesised that the reduced PARPi sensitivity observed in ATM-deficient cells upon loss of SETD1A may also be due to restoration of HR.

**Fig. 2:**
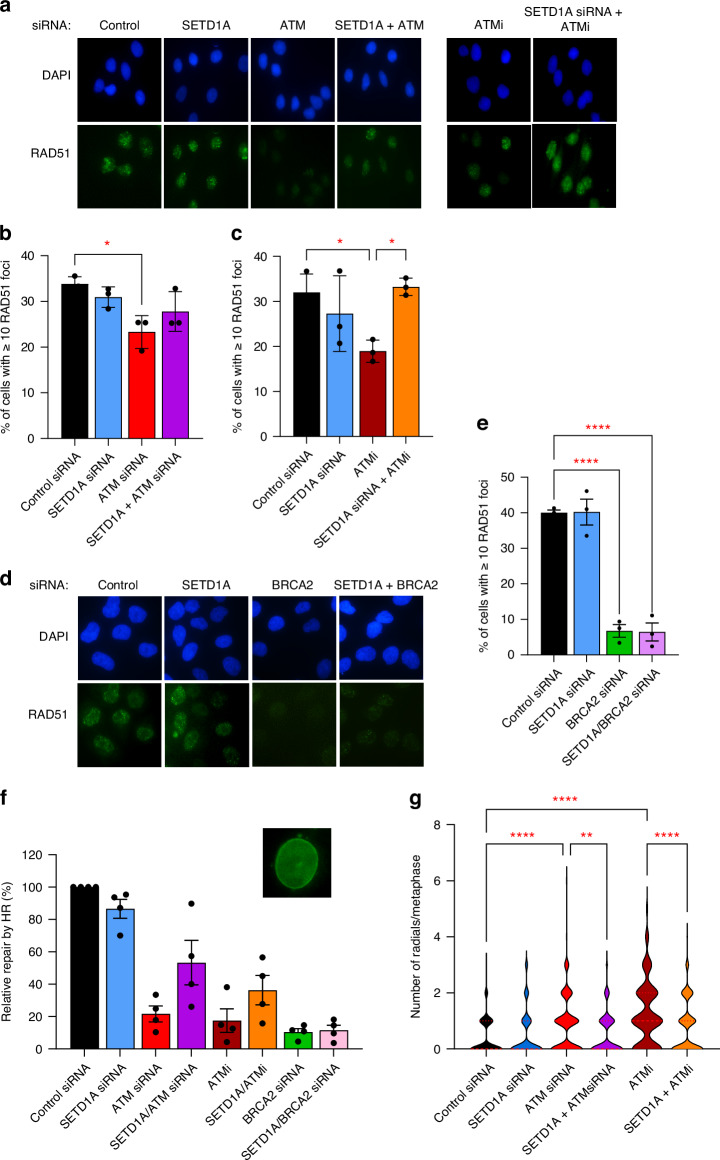
Loss of SETD1A partially restores homologous recombination (HR) activity in ATM-deficient cells treated with Olaparib. **a**–**c** HeLa cells were transfected with the indicated siRNAs and/or treated with 1 µM AZD0156 for 24 h as indicated, and then treated with 5 µM Olaparib for a further 24 h. Cells were immunostained with an antibody against RAD51 and nuclei counterstained with DAPI, and foci enumerated. Representative images are shown in (**a**), and data from 3 independent biological repeats shown in (**b**, **c**). **d**, **e** MCF-7 cells were transfected with the indicated siRNAs, and treated with 5 µM Olaparib for 24 h. Cells were immunostained as above: representative images are shown in (**d**), and data from 3 independent biological repeats shown in (**e**). **f** HeLa cells were transfected/treated as denoted, left for 24 h, and then transfected with CRISPR based LMNA-HR plasmids encoding Cas9 nickase, a fluorescent Clover-Lamin and a plasmid encoding red fluorescent protein (RFP). Percentage HR was calculated by the number of fluorescent Lamin-expressing cells normalised to RFP expression. **g** Cells from F were incubated with 5 µM Olaparib for 24 h, and the number of radial chromosomes per metaphase analysed by DAPI staining and quantified. In all cases data represent the mean ± SEM from at least three independent experiments. * = *P* < 0.05, ** = *P* < 0.01 *** = *P* < 0.0005, **** = *P* < 0.0001 as determined using a one-way ANOVA with post-hoc Tukey test for multiple comparisons.

To examine this, we first assessed RAD51 foci formation as a marker of HR following Olaparib treatment. Knockdown or inhibition of ATM resulted in a significant defect in RAD51 foci formation (Fig. 2a–c). In both cases, depletion of SETD1A increased RAD51 foci formation to near-normal levels, which was most notable in the presence of ATMi (Fig. 2c). In agreement with our findings above, loss of SETD1A from BRCA2-depleted MCF-7 cells had no restorative effect on RAD51 foci formation (Fig. 2d, e). To assess HR activity directly, we utilised a CRISPR-based assay in which cells utilise Clover-tagged Lamin as a template for functional HR, resulting in green fluorescent-ringed nuclei [39]. Using this assay, ATM knockdown or inhibition significantly decreased HR, whilst co-depletion of SETD1A from these cells resulted in partial HR restoration (Fig. 2f and Fig. S1C).

PARP inhibition in HR-deficient cells is also characterised by the NHEJ-mediated formation of chromosomal radials which drive cell death. We therefore set out to confirm whether toxic end-joining was decreased following SETD1A depletion in ATM-deficient cells, using metaphase spreads to quantify radial chromosome formation. Consistent with the data above, cells lacking ATM displayed elevated toxic radial formation upon Olaparib treatment, whilst co-depletion of SETD1A reduced this back to control levels (Fig. 2g and Fig. S1D). This provides further evidence that loss of SETD1A results in a shift in pathway usage from NHEJ towards HR, thus restoring recombination in certain HR-deficient backgrounds.

In addition, the ability of PARPi to trap PARP1/2 onto damaged chromatin correlates with their potency in vitro [48]. We postulated that resistance to Olaparib upon loss of SETD1A might additionally reduce PARP1/2 trapping, leading to decreased cell death. Surprisingly however, levels of PARP1 on chromatin bore little correlation with sensitivity to PARPi (Fig. S2): although both ATM deficiency and SETD1A deletion *via* Cas9 editing decreased PARP1 trapping after cell fractionation, they had opposite phenotypic effects. This strongly suggested that reduced PARP trapping was not responsible for PARPi sensitivity/resistance in these cells. Instead, our findings demonstrate that PARPi resistance upon loss of SETD1A arises due to loss of pro-NHEJ functions and restoration of HR.

### Perturbing H3K4 methylation reduces sensitivity of ATM-deficient cells to Olaparib by partially restoring HR

**Fig. 3:**
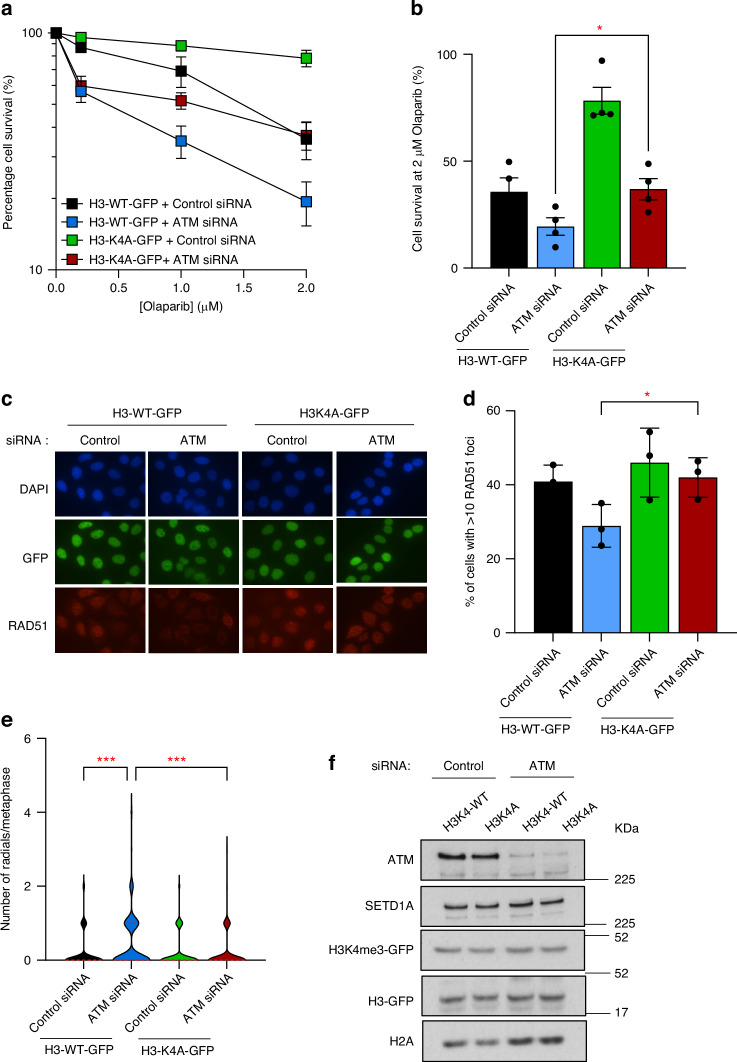
Loss of H3 lysine 4 methylation reduces sensitivity of ATM-deficient cells to Olaparib and partially restores homologous recombination. **a**, **b** H3K4-WT-GFP or H3K4A-GFP Hela cells were transfected with the indicated siRNAs and left to form colonies under continual exposure to Olaparib at the denoted doses for 10 days. **b** denotes relative cell survival at 2 µM Olaparib. Data represent mean ± SEM from four independent repeats. **c**, **d** H3K4-WT-GFP or H3K4A-GFP cells from (**a**) were treated with 5 µM Olaparib for 24 h, immunostained with antibodies against RAD51, and foci enumerated. Representative images are shown in (**c**), and data from 3 independent biological repeats shown in (**d**). **e** Cells from (**a**) were incubated with 5 µM Olaparib for 24 h, and the number of radial chromosomes per metaphase analysed by DAPI staining and quantified. **f** Whole cell extracts of cells from (**a**) were analysed by immunoblotting using the indicated antibodies. In all cases, data represent mean ± SEM from > 3 biologically independent experiments. * = *P* < 0.05 as determined by an unpaired two-tailed Students *t* test (**b**, **d**), *** = *P* < 0.0005 as determined by a one-way ANOVA with post-hoc Tukey test for multiple comparisons (**e**).

SETD1A catalyses mono-, di- and tri-methylation of lysine 4 on histone H3 (H3K4), protecting stalled replication forks and promoting NHEJ [22, 37, 49]. To investigate whether H3K4me3 was directly responsible for our observations in *ATM*-deficient backgrounds, we used a mutant cell line expressing either wild-type GFP-tagged histone H3 or a H3K4A mutant which perturbs methylation at lysine 4, and that phenocopies loss of SETD1A [37, 38]. Using these cells, we examined the impact of Olaparib treatment in the presence/absence of ATM. In agreement with our previous observations, ATM deficiency sensitised H3-WT-GFP cells to Olaparib and substantially compromised HR. In contrast, depletion of ATM only partially sensitised H3-K4A-GFP cells to PARPi (Fig. 3a, b), and these cells showed increased levels of RAD51 foci (Fig. 3c, d) and a reduction in the formation of toxic chromosomal radials (Fig. 3e, f). In combination with previous findings, this suggests that loss of SETD1A-mediated H3K4 methylation influences PARPi sensitivity by restoring HR.

### Loss of SETD1A reduces sensitivity of HR-deficient cancer cell lines to Olaparib

**Fig. 4:**
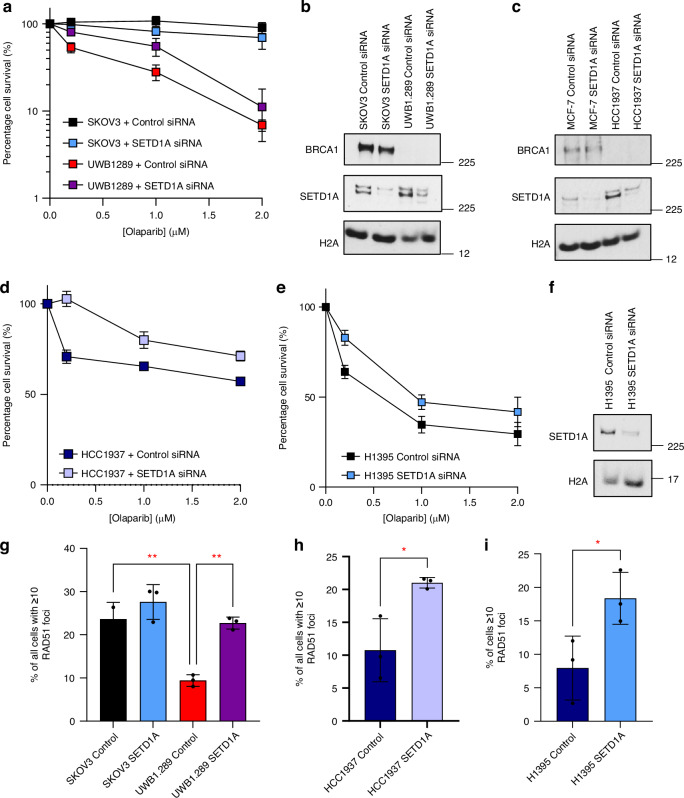
Loss of SETD1A reduces sensitivity of HR-deficient cancer cell lines to Olaparib via partial restoration of HR. **a**–**f** SKOV3, UWB1.289, MCF-7, HCC1937 or H1395 cells were transfected with the indicated siRNAs. Cells were plated at low density and left to form colonies under continual exposure to Olaparib at the denoted doses for 10 days (**a**, **d**, **e**). Whole cell extracts were analysed by immunoblotting in (**b**, **c**, **f**). **g**–**i** Transfected cells from (**a**, **d**, **e**) were treated with 5 µM Olaparib for 24 h, and immunostained with antibodies against RAD51. RAD51 foci were enumerated and shown in (**g**–**i**). All data represent the mean ± SEM from >3 independent experiments. * = *P* < 0.05, ** = *P* < 0.01 *** = *P* < 0.0005, **** = *P* < 0.0001 as determined a one-way ANOVA with post-hoc Tukey test for multiple comparisons.

We next wanted to extend our findings from HeLa cells into cancer cell lines bearing pathogenic *BRCA1* or *ATM* mutations. We therefore selected 3 cell lines encoding *BRCA1* (UWB1.89 and HCC1937) or *ATM* (H1395) mutations, with non-mutated SKOV3, MCF-7 and A549 cell lines as cancer-type matched controls. As expected, *BRCA1*-mutated UWB1.289 ovarian cancer (Fig. 4a, b) and HCC1937 breast cancer cells (Fig. 4c, d) displayed significant sensitivity to Olaparib. In both cases, depletion of SETD1A reduced the sensitivity of *BRCA1*-mutated cell lines to Olaparib but had no effect on *BRCA1*-proficient SKOV3 or MCF-7 cells (data not shown). Similar results were obtained in *ATM*-mutated H1395 non-small cell lung cells, where SETD1A depletion again compromised PARPi sensitivity (Fig. 4e, f), which was not observed in *ATM* wild-type A549s (data not shown). Moreover, and in agreement with preceding data, loss of SETD1A also restored Olaparib-induced RAD51 foci formation and thus HR activity in both *BRCA1*-mutated ovarian and breast cancer cell lines and in *ATM*-mutated lung cancer cells (Fig. 4g–i). In concert, these findings firmly establish that loss of SETD1A drives PARPi resistance in breast, ovarian and lung cancer cells by restoring HR.

### SETD1A-dependent transcription of EME1 drives PARPi resistance

**Fig. 5:**
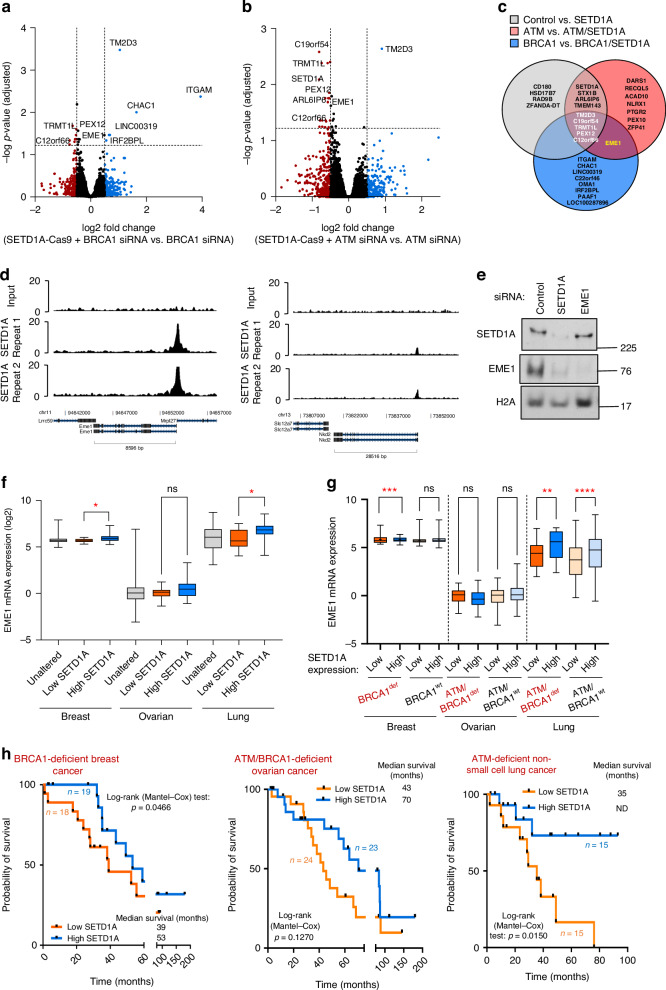
SETD1A-dependent EME1 transcription in ATM- and BRCA1-deficient cells. **a**, **b** Inducible Hela Kyoto iCas9 cells expressing SETD1A gRNA were treated with doxycycline and transfected with the indicated siRNAs. Gene expression was analysed using RNA-seq and differential gene expression displayed as a volcano plot. Significantly downregulated genes are represented in red and significantly upregulated genes denoted in blue. Cutoff values for differentially expressed genes were Log2 fold change = 0.5 and −log (adjusted P-value) = 1.22. Data points represent means from 3 independent repeats. **c** Venn diagram of overlapping differentially expressed genes from (**a**, **b**). **d** Chromatin immunoprecipitation profiles of murine Setd1a over the TSS of genes from (**c**). Source data are from [44]. **e** Whole cell extracts of Hela Kyoto iCas9 cells expressing SETD1A gRNA were analysed by immunoblotting using the denoted antibodies. **f** Log2 mRNA expression of EME1 in breast, lung or ovarian carcinoma patients triaged by SETD1A expression. **g** EME1 expression data from (**f**) was triaged into BRCA1- or ATM/BRCA1-deficient patient populations. **h** Kaplan–Meir curves denoting overall survival for the indicated cancer patient subtypes stratified by low (lower 50%) or high (upper 50%) SETD1A mRNA expression. * = *P* < 0.05, *** = *P* < 0.0005, as determined by a one-way ANOVA with post-hoc Tukey test for multiple comparisons (**f–g**). In (**f**–**h**), Low= homozygous SETD1A deletion or mRNA expression <= -2 SD below mean; high= SETD1A mRNA expression >2 SD above mean. Statistical significance of the data in **h** was determined by a Log-rank (Mantel-Cox) test.

Given that SETD1A-mediated H3K4me plays a key role in transcriptional regulation [44, 49], we postulated that gene expression changes may account for our observations. To investigate this, we utilised an established isogenic HeLa cell system that expressed a gRNA targeting the *SETD1A* locus, in which SETD1A could be deleted by inducing Cas9 expression (HeLa Kyoto iCas9; Fig. S3). These cells phenocopied those depleted of SETD1A using RNAi, including defective recruitment of RIF1 to damaged chromatin (Fig. S3A-C). We then depleted either ATM or BRCA1 and performed RNA sequencing (RNA-seq) analysis to determine differentially expressed (DEGs) regulated by SETD1A (Fig. 5a, b and Fig. S3E-H). Surprisingly, loss of SETD1A had a limited impact on gene expression in HR-proficient cells, with only 13 transcripts showing significant deregulation upon Cas9 expression. To identify genes deregulated in PARPi-sensitive or resistant contexts, we compared DEGs between *BRCA1*-deficient, *ATM*-deficient, and HR-proficient backgrounds in the presence/absence of SETD1A. This identified 5 genes whose expression was commonly regulated by SETD1A irrespective of HR proficiency (denoted in white in Fig. 5c), and one gene, EME1, that was downregulated in different HR-deficient PARPi-resistance backgrounds (indicated in yellow in Fig. 5c). Importantly, these gene expression changes were validated using qPCR in additional replicate samples from each cell type, except for PEX12 (Fig. S4). By analysing mouse ChIP-seq datasets [44], we also observed that SETD1A was highly enriched at the TSS of EME1, but not at the TSS of genes regulated by BRCA1 or ATM (Fig. 5d and Fig. S5). Finally, we confirmed our findings at the protein level, where SETD1A deletion substantially decreased EME1 protein expression (Fig. 5e). Overall, these analyses suggests that whilst the transcription of only a small number of genes is dependent on SETD1A in our iCas9 model, down-regulation of EME1 might represent a common mechanism linked to PARPi resistance.

### Deregulated gene expression in cancer patients with altered SETD1A expression

We next set out to investigate the relevance of our findings using cancer genomics data [41, 42]. We first stratified lung adenocarcinoma, breast carcinoma or ovarian carcinoma patients by SETD1A mRNA expression and then assessed levels of each DEG. Consistent with our previous analyses, patients with low SETD1A levels (<-2 SD log2 mRNA expression) had decreased expression of DEGs identified as downregulated by RNA-seq, including EME1 (Fig. 5f and Fig. S6). This was also observed in HR-deficient breast and lung cancer cohorts (Fig. 5g) and cancer cell lines (Fig. S7A). We next investigated whether these observations affected clinical outcome by examining overall survival. Firstly, we identified cohorts of HR-deficient breast, ovarian and lung cancer patients [41, 42], stratified them by high/low SETD1A mRNA expression as before, and analysed overall survival rates. This revealed that *BRCA1*- and *ATM*-deficient cancer patients with low SETD1A mRNA expression had poorer overall survival outcomes compared to those with higher levels of SETD1A (Fig. 5h). This was not the case for BRCA2-deficient patients, nor was it the case for HR-proficient patients (Fig. S7B, C). Low SETD1A levels also correlated with decreased progression-free survival in these HR-deficient patients (Fig. S7D, E), and with lower tumour mutational burdens (Fig. S7F).

Finally, we assessed whether EME1 might correlate with patient survival in a similar way to SETD1A. Interestingly, in most cases, EME1 expression did not correlate with increased/decreased patient survival, except in the case of BRCA1-deficient breast cancer, where low EME1 expression associated with a favourable prognosis (Fig. S7G–J), although these two factors had partially separable impacts on survival (Fig. S7K). Together, this demonstrates that expression of SETD1A, and to a limited extent of EME1, correlates with survival of HR-deficient patients.

### Loss of EME1 drives PARPi resistance in HR-deficient cells by restoring HR

**Fig. 6:**
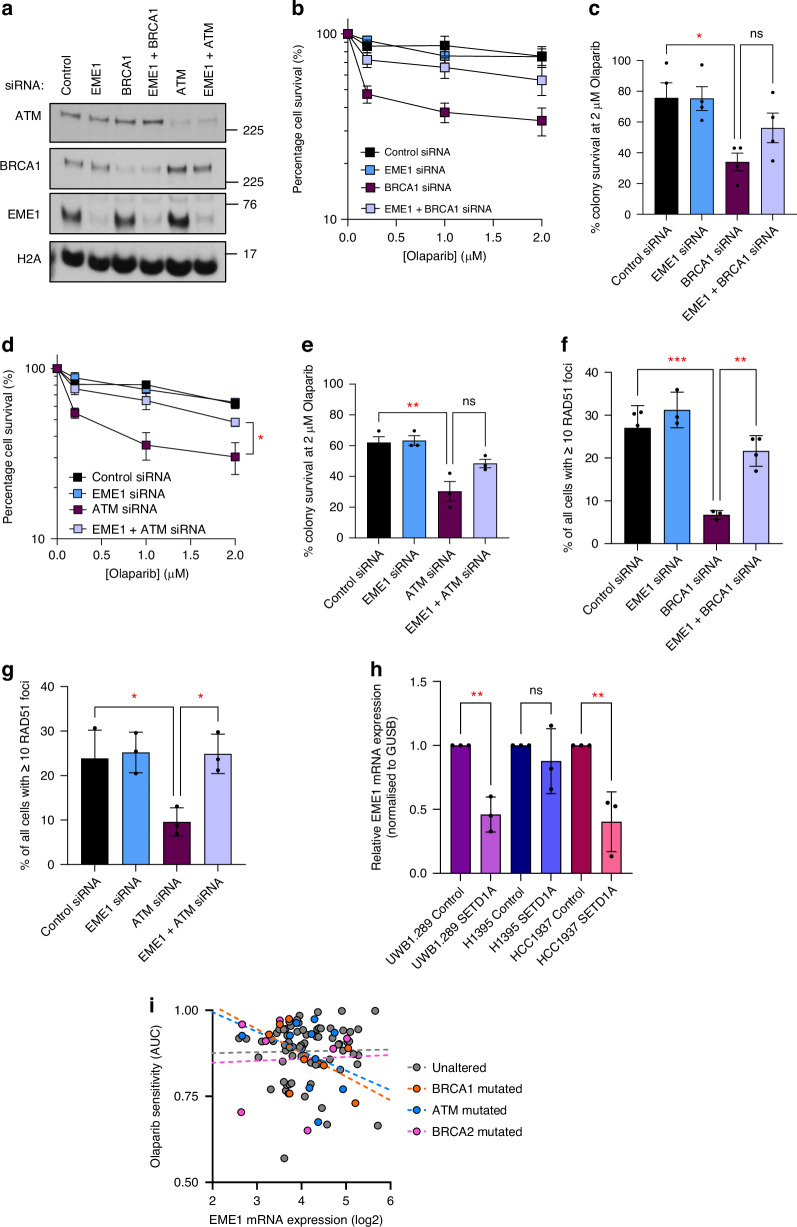
Deregulated EME1 expression partially restores HR and drives resistance of Olaparib in ATM- and BRCA1-deficient cells. **a**–**e** HeLa cells were transfected with the indicated siRNAs for 48 h. Whole cell extracts were analysed by immunoblotting (**a**). Cells were plated at low density and left to form colonies under continual exposure to Olaparib at the denoted doses for 10 days (**b,** **d**). **c**, **e** denotes relative cell survival at 2 µM Olaparib. **f**, **g** Cells from (**a**) were treated with 5 µM Olaparib for 24 h, immunostained with antibodies against RAD51, and foci enumerated. **h** mRNA was isolated form cells from (**a**), and EME1 and GUSB mRNA expression levels determined by qPCR. **i** Olaparib sensitivity data from DepMap [43] triaged into unaltered and mutated groups as denoted. Dotted lines = linear regression. In all cases except panel **i**, data points represent the mean ± SEM from 3 independent repeats. * = *P* < 0.05, ** = *P* < 0.01, *** = *P* < 0.0005, **** = *P* < 0.0001 as determined by a two-tailed unpaired Students *t* test.

Given that SETD1A promotes EME1 transcription in vitro, and that this correlation was observed in cancer genomic datasets, we hypothesised that EME1 downregulation might drive the PARPi resistance observed in the absence of SETD1A. To assess this independently of SETD1A, we depleted EME1 and/or BRCA1/ATM from HeLa cells (Fig. 6a) and assessed the response to Olaparib. Excitingly, EME1 loss phenotypically mirrored SETD1A depletion in both these backgrounds *i.e*. its loss compromised PARPi sensitivity (Fig. 6b–e). Surprisingly, given its known roles in later stages of HR where it processes recombination intermediates [50–52], loss of EME1 also restored levels of HR in both *ATM-* or *BRCA1-*deficient cells after exposure to PARPi (Fig. 6f, g), precisely as observed with SETD1A loss. Furthermore, downregulation of EME1 was also observed when SETD1A was depleted from *BRCA1*-mutated HCC1937 breast cancer cells, or *ATM*-mutated H1395 lung cancer cells (Fig. 6h), suggesting that this represents a conserved mechanism in multiple cancer types. Indeed, in *BRCA1*- or *ATM*-mutated breast, ovarian and lung cancer lines, low EME1 levels associated with decreased Olaparib sensitivity (Fig. 6i), which was not the case in *BRCA2-*mutated or unaltered cells. Together, this demonstrates that loss of SETD1A-dependent EME1 transcription drives PARPi resistance in HR-deficient cells.

In summary, we show that SETD1A loss renders *ATM*-deficient and *BRCA1*-mutated breast, lung, and ovarian cancer cells resistant to PARPi by restoring HR, and that this is partly driven by defective EME1 transcription.

## Discussion

Multiple mechanisms of PARPi resistance in BRCA-deficient cells have now been described and have highlighted clear differences between resistance arising in *BRCA1*- and *BRCA2*-mutated cancers e.g. [8, 53, 54]. However, there have been only limited numbers of studies examining PARPi resistance in cells lacking other HR factors. This, coupled with mixed reports of PARPi efficacy in trials in other cancer backgrounds of HR-deficiency [31, 33–36, 55–58], led us to examine whether loss of the lysine methyltransferase and pro-NHEJ factor SETD1A influenced PARPi efficacy outside BRCA1.

Here we show that loss of SETD1A also confers PARPi resistance in *ATM*-deficient cancer cells. Inhibition of ATM activity or depletion of ATM sensitised cervical cancer lines to Olaparib, in line with numerous previous studies demonstrating that ATM deficiency correlates with PARPi sensitivity [24–27, 59–62]. However, depletion of SETD1A rendered these cells at least partially resistant to PARPi (Fig. 1). Moreover, we observed similar resistance in *ATM*-mutated H1395 NSCLC cells depleted of SETD1A (Fig. 4), demonstrating that this phenomenon was not cell-type specific or associated with ATM inhibitor or depletion using siRNA. Although the extent of PARP1 trapping by Olaparib was not affected by SETD1A depletion in these models (Fig. S2), we observed restoration of HR by RAD51 foci, Cas9-nickase mediated recombination and decreased toxic chromosome radials (Figs. 2 and 4). This is entirely consistent with our previous findings that SETD1A depletion restored HR in *BRCA1*-deficient cells [22, 23]. It also suggests that a deficiency in resection caused by loss of ATM can be overcome by compromising the 53BP1-RIF1-Shieldin pathway. This agrees with studies in *ATM-*deficient breast cancer cells showing that 53BP1 depletion causes PARPi resistance [47], but is at odds with the demonstration that loss of 53BP1 cannot suppress the PARPi sensitivity of Atm^*-/-*^ mESCs [63]. Whilst it is difficult to reconcile these differences, it may be that the genetic background and/or tumoral nature of MCF-7, CAL51, HeLa or H1395 cells used in our study or that of Hong and colleagues [47] is responsible. In addition, the necessity for ATM in activating the 53BP1-RIF1 axis *via* 53BP1 phosphorylation likely plays an important role [13, 15]. Nevertheless, our findings strongly suggest that perturbation of pro-NHEJ pathways by loss of SETD1A drives PARPi resistance.

Our findings also demonstrate that loss of SETD1A represents a conserved mechanism of PARPi resistance across *BRCA1-* and *ATM-*deficient cancer cells (this study and [22]) but this is not extended to cells lacking *BRCA2* (Figs. 1 and 2). Moreover, the survival of *BRCA1-* or *ATM-*deficient cancer patients could be triaged by SETD1A expression, which was not the case for those with deficiencies in *BRCA2*, nor those with functional copies/levels of these genes (Fig. S6). These observations are likely due to the differing roles of ATM, BRCA1 and BRCA2 in HR. Whilst ATM and BRCA1 promote the initial steps of HR and resection and their loss can partially be overcome by loss of competing pathways, BRCA2 is absolutely required for RAD51-dependent recombination. This is in keeping with multiple studies demonstrating that loss of pro-NHEJ factors cannot drive PARPi resistance in *BRCA2*-deficient cells [53, 54].

Interestingly, SETD1A-dependent methylation of H3K4 and subsequent transcription also seems to underpin PARPi resistance in HR-deficient cells lacking this KMT. Firstly, perturbation of H3K4me using our H3-K4A-GFP system phenocopied SETD1A loss, alleviating PARPi sensitivity, restoring HR and decreasing PARPi-induced toxic radial formation in the absence of ATM (Fig. 3). Moreover, RNAseq analyses revealed that expression of EME1 mRNA was tightly correlated with PARPi resistance, with SETD1A-depleted cells displaying ~2-fold reduction in EME1 mRNA and protein levels (Fig. 5 and Fig. S5). In addition, SETD1A was enriched at the TSS of EME1 in mESCs but was absent from genes regulated by either BRCA1 or ATM, suggesting both specificity and a wider role for SETD1A in transcriptionally regulating EME1. Moreover, loss/perturbation of EME1 phenocopies SETD1A loss in the absence of both BRCA1 and ATM and led to the restoration of HR (Fig. 6), which was supported by data from cancer cell lines. EME1 forms part of the SLX-MUS81-EME1 structure-specific nuclease that plays a key role in resolving Holliday junctions, including during the latter stages of HR [44, 50–52, 64]. Our findings are thus in broad agreement with findings from the D’Andrea lab [65] that loss of MUS81 renders BRCA2-deficient cells resistant to PARPi, but in conflict with other reports. However, our data also suggest a new anti-recombinogenic role for EME1 in HR-deficient cells. Whether this involves other members of this nuclease complex, and whether the replication stress-specific paralog EME2 [66] is also implicated, remains to be determined.

Previously, we attributed the PARPi resistance in *BRCA1*-deficient cells lacking SETD1A to a failure to recruit RIF1, arising from defective H3K4 methylation and a subsequent loss of binding between H3K4me3 and RIF1 at damaged sites [22]. Here, we offer an additional mechanism controlled by SETD1A/H3K4me that influences PARPi sensitivity: the transcription of EME1. Although this appears contradictory, this mirrors the multiple functions of other DNA repair proteins that influence PARPi response, for example the roles of BRCA1 in HR, fork protection, and ssDNA gap formation [8–10, 67, 68]. The respective contributions of these mechanisms to PARPi resistance, or whether these SETD1A-dependent mechanisms are interlinked, are clearly areas for future study.

Finally, these findings reinforce our previous speculation [23] that SETD1A status/activity could be a useful prognostic biomarker to monitor/predict PARPi response. Indeed, SETD1A expression correlates with overall survival in HR-deficient breast, ovarian and lung cancers (Fig. 5). It is plausible that monitoring SETD1A levels or using EME1 expression or H3K4me3 as proxies for its activity might be useful to predict PARPi resistance, although this warrants closer investigation. It is tempting to speculate that this may ultimately improve the use of PARPi such as Olaparib in cancers where its efficacy has been limited (e.g. NSCLC [36]), by identifying patients amenable for therapy.

## Supplementary information

Supplementary Material

## Data Availability

All original data is archived and stored at the University of Birmingham via UBIRA eData which can be searched at https://edata.bham.ac.uk/. RNAseq data is available at the NCBI Gene Expression Omnibus (https://www.ncbi.nlm.nih.gov/gds/) using the accession number GSE271430. HeLa-iCas9-SETD1A gRNA cell lines are available upon request to the corresponding author.
